# The Dichotomous Pattern of IL-12R and IL-23R Expression Elucidates the Role of IL-12 and IL-23 in Inflammation

**DOI:** 10.1371/journal.pone.0089092

**Published:** 2014-02-21

**Authors:** Gaëlle Chognard, Lisa Bellemare, Adam-Nicolas Pelletier, Maria C. Dominguez-Punaro, Claudine Beauchamp, Marie-Josée Guyon, Guy Charron, Nicolas Morin, Durga Sivanesan, Vijay Kuchroo, Ramnik Xavier, Stephen W. Michnick, Sylvain Chemtob, John D. Rioux, Sylvie Lesage

**Affiliations:** 1 Research Center, Maisonneuve-Rosemont Hospital, Montréal, Québec, Canada; 2 Département de Microbiologie, Infectiologie et Immunologie, Université de Montréal, Montréal, Québec, Canada; 3 Research Center, Montreal Heart Institute, Montréal, Québec, Canada; 4 Département de Biochimie, Université de Montréal, Montréal, Québec, Canada; 5 Center for Neurologic Diseases, Brigham and Women’s Hospital, Harvard Medical School, Boston, Massachusetts, United States of America; 6 Division of Medical Sciences, Harvard University, Boston, Massachusetts, United States of America; 7 Departments of Pediatrics, Ophthalmology, and Pharmacology, Centre Hospitalier Universitaire Ste-Justine Research Center, Montréal, Québec, Canada; 8 Département de Médicine, Université de Montréal, Montréal, Québec, Canada; Heart Research Institute, Australia

## Abstract

IL-12 and IL-23 cytokines respectively drive Th1 and Th17 type responses. Yet, little is known regarding the biology of these receptors. As the IL-12 and IL-23 receptors share a common subunit, it has been assumed that these receptors are co-expressed. Surprisingly, we find that the expression of each of these receptors is restricted to specific cell types, in both mouse and human. Indeed, although IL-12Rβ2 is expressed by NK cells and a subset of γδ T cells, the expression of IL-23R is restricted to specific T cell subsets, a small number of B cells and innate lymphoid cells. By exploiting an IL-12- and IL-23-dependent mouse model of innate inflammation, we demonstrate an intricate interplay between IL-12Rβ2 NK cells and IL-23R innate lymphoid cells with respectively dominant roles in the regulation of systemic *versus* local inflammatory responses. Together, these findings support an unforeseen lineage-specific dichotomy in the *in vivo* role of both the IL-12 and IL-23 pathways in pathological inflammatory states, which may allow more accurate dissection of the roles of these receptors in chronic inflammatory diseases in humans.

## Introduction

The heterodimeric receptors for both IL-12 and IL-23 share a common protein subunit, namely IL-12Rβ1, and are thus often depicted at the same cell membrane [1]–[5]. IL-12Rβ2 and IL-23R, the respective specific subunits of IL-12 and IL-23 receptors, show high homology and likely arose by gene duplication [1]. This suggests a possible coordination for the expression of both IL-12 and IL-23 receptors [1]. Yet, the expression pattern of the receptors for IL-12 and IL-23 has not been defined.

A better comprehension of the biology of the receptors for IL-12 and IL-23 is essential, as both pathways are involved in chronic inflammatory diseases [6]–[9]. *IL23R* was first discovered to have a role in human disease as a result of one of the first published GWA studies of a complex trait. Specifically, it was demonstrated that the Glu allele at residue 381 in the IL-23R protein conferred significant protection against developing Crohn’s disease and ulcerative colitis, while other genetic variants in this gene, in contrast, conferred increased risk [6]. Recent targeted deep re-sequencing experiments in Crohn’s disease patients and healthy controls have identified further protective variants in the *IL23R* [10], [11]. In an association study of psoriasis, it was shown that the 381Glu allele had a similar protective effect, consistent with the clinical observations that psoriasis and Crohn’s disease are co-segregated with high frequency [12]. Further supporting a central role of this pathway in chronic inflammatory disease susceptibility are the confirmed associations of the *IL12B* gene (encoding the p40 subunit of both IL-12 and IL-23 cytokines) with Crohn’s disease, ulcerative colitis and psoriasis; the *JAK2* gene (a proximal kinase in the IL-23R pathway) in Crohn’s disease; and the *STAT3* gene (immediately downstream of *JAK2*) in Crohn’s disease and ulcerative colitis [13], [14]. Similarly, genetic polymorphisms in *IL12RB2* are also associated with increased risk of chronic inflammatory disease. Notably, a recent GWA study of primary biliary cirrhosis revealed an association with *IL12RB2*, *IL12A* (a gene encoding for the p35 subunit of the IL-12 cytokines) and *STAT4* (downstream of *IL12RB2*) [9], [15]. Genetic polymorphisms in both the IL-12 and IL-23 pathways are thus strongly associated with susceptibility to inflammatory diseases. Moreover, these pathways are current molecular targets in the treatment of chronic inflammatory diseases. It is thus imperative that we understand the cellular players of the IL-12 and IL-23 pathways and their role in inflammation.

In this study, we demonstrate that, although they share a common subunit, IL-23 and IL-12 receptors are not expressed on the same cell populations. We establish that there is a strong dichotomy between IL-12 and IL-23 receptor expressing cells, which is quite conserved in humans and mice. We additionally exploit an IL-12 and IL-23 dependent model to demonstrate that there is an intricate interplay between the IL-12 and IL-23 pathways in regulating both systemic and local innate inflammatory responses.

## Materials and Methods

### Ethics Statement

The work with human samples was approved by the Montreal Heart Institute Research Ethics Board (#12–1363). Peripheral blood was obtained from healthy volunteers after obtaining written informed consent. The mouse studies were approved by the Maisonneuve-Rosemont Hospital ethics committee and overseen by the Canadian Council for Animal Protection.

### PBMCs

PBMCs were obtained by centrifugation of collected blood in BD Vacutainer® CPT Cell Preparation Tube with Sodium Citrate, and washed with DPBS for platelet removal. CD14+ monocytes were enriched from PBMCs with CD14 MicroBeads (MACS Miltenyi Biotec) on LS columns. Both CD14- and CD14+ fractions were treated with FcR blocking reagent (MACS Miltenyi Biotec) and further stained for cell sorting.

### Mice

Eight- to 12 week-old mice were used for all the experiments. C57BL/6J mice (#664), IL-12Rβ2^−/−^ (#3248), RORγt^−/−^ (#7572) and Rag1^−/−^ (#2216) on C57BL/6 background were purchased from the Jackson Laboratory. IL-12Rβ2^−/−^, IL-23R-eGFP^+/−^ and IL-23R-eGFP homozygous mice [16] (referred to in the text as IL-23R^−/−^) were maintained on a C57BL/6 background. IL-23R^−/−^ and IL-12Rβ2^−/−^ were bred to Rag1^−/−^ to respectively generate IL-23R^−/−^.Rag1^−/−^ and IL-12Rβ2^−/−^.Rag1^−/−^ mice. All mice were maintained at the Maisonneuve-Rosemont Hospital housing facility (Montreal, Canada).

### Mouse Cell Isolation

Spleens, lymph nodes, and thymus were treated with collagenase (1 mg/ml; type V from Clostridium histolyticum; Sigma-Aldrich) for 15 min at 37°C. Bone marrow was flushed from the femur and tibias. Lungs were perfused with PBS, harvested, and digested for 30 min at 37°C with 1 ml collagenase. Intestines were collected in cold media and kept on ice prior to treatment. They were then placed on a shaker at 450 rpm for 20 minutes at 37°C in RPMI medium containing 30% FBS, 5 mM EDTA and 145 µg/mL DTT. Following this incubation, the intestines were hand-shaken in RPMI containing 2 mM EDTA and filtered through a kitchen strainer. Intestines were then cut in 1 cm fragments and placed in a shaker at 450 rpm for 30 minutes at 37°C with RPMI containing 50 µg/mL liberase, 14.5 µg/mL DNase I and 135 µg/mL collagenase V. After digestion, cell suspensions of the lamina propria are obtained by pressing the tissue fragments through a 100 µm sterile cell strainer (BD BioSciences, Mississauga, Canada). All organs were finally passed through a 70-µm cell strainer (BD Biosciences, Mississauga, Canada) to yield single-cell suspensions prior to staining with antibodies. NH_4_Cl was used for erythrocyte lysis of single cell suspensions on the spleen, bone marrow and lung. Cell counts were performed by trypan blue exclusion using a hemacytometer. Three million cells were stained with different combinations of fluorochrome-conjugated antibodies.

### Flow Cytometry

Human CD14- PBMC fraction was stained with antibodies to the following markers: CD3 APC-Cy7 (clone UCHT1), CD4 FITC (clone RPA-T4), CD8α APC (clone RPA-T8), TCR γδ biotin (clone B1), streptavidin PE-Cy5 and CD19 PE (clone HIB19) from Biolegend; CD56 PE-Cy7 (clone B159) from BD Biosciences. Human CD14+ monocyte-enriched fraction was counterstained with goat anti-mouse IgG2a FITC (AbD Serotec) to bind bead-coupled monoclonal anti-human CD14 (mouse IgG2a). Cell sorting was performed on a FACS Aria III (BD Biosciences). Electronic gating was performed on CD14- PBMC fraction to sort B cells (CD3- CD19+), CD4 T cells (CD3+ CD4+), CD8 T cells (CD3+ CD8+), γδ T cells (CD3+ TCR γδ+) and NK cells (CD3- CD19- CD56+), and on CD14+ monocyte-enriched fraction to sort monocytes (FSC/SSC gating and IgG2a+). Cell sorting was performed on a FACS Aria III (BD Biosciences).

Murine cells were stained with antibodies to the following markers: CD3ε PE and APC (clone 145.2C11), CD8α PE (clone 53-6.7), CD11b Pacific Blue (clone M1/70), CD11c PE and PE-Cy7 (clone N418), CD19 PerCP (clone 6D5), CD27 PE (clone LG.3A10), CD45 PE-Cy7 (clone 30-F11), B220 (CD45R) Alexa700 (clone RA3-6B2), CD49b Pacific Blue (clone DX5), CD90.2 PerCP (clone 30-H12), CD117 APC (clone 2B8), CD127 Biotin (clone A7R340), TCRγδ PerCP-Cy5.5 (clone GL3), TCRβ Alexa700 (clone H57-597), NK1.1 APC (clone PK136), Gr1 PerCP (clone RB6-8C5), from Biolegend; Sca1 PE-Cy7 (clone D7), IL12Rβ2 (clone HAM10B9), anti-hamster IgG PE (clones G70-204, G94-90.5) from BD Biosciences; Streptavidin Alexa700 from Invitrogen; NKp46 PE (clone 29A1.4), CD4 PE-Cy7 (clone GK1.5) from eBioscience; and mouse pDC antigen-1 APC (clone JF05-1C2) from Miltenyi Biotec; IL12Rβ2 APC, PE (clone 305719) from R&D Systems. Cell viability was assessed using Viability dye eFluor 780 from eBioscience. For analyzing IL23R-eGFP positive cells, flow cytometry data collection was performed on an LSRII (BD). Files were analyzed using FlowJo software (TreeStar, Inc). Doublet cell exclusion was performed by electronic gating based on FSC and SSC profiles. Cell sorting was performed on a FACS Aria III (BD Biosciences). Electronic gating was performed to sort B cells (CD19+ B220+), CD4 T cells (TCRβ+ CD4+), CD8 T cells (TCRβ+ CD4+), γδ T cells (TCR γδ+) and NK cells (CD49b+ B220-). For specific populations of γδ T cells, electronic gating was performed to sort CD27+ and CD27- TCR γδ+ cells.

### RNA Isolation, cDNA Preparation, and RT-PCR

mRNA from sorted human PBMC or mouse spleen cells was extracted using RNeasy Mini Plus or Micro Plus kits, respectively for more or less than 500 000 cells, according to the manufacturer’s instructions. Human mRNA was treated with DNaseI according to manufacturer instructions. After storage at 4°C in RNAlater (Qiagen), mRNA from proximal colon was isolated by using mechanical homogenization with Tissue Lyser (Qiagen) followed by extraction with RNeasy Mini Plus kit according to the manufacturer’s instructions. Total RNA was quantified on RNA 6000 Nano chips on a 2100 Bioanalyzer (Agilent Technologies) or on Tecan’s NanoQuant Plate on Infinite Reader. cDNA was generated using High-Capacity cDNA Reverse Transcription kit (Applied Biosystems). RT-PCR was performed on cDNA using SYBR green chemistry (Applied Biosystems) and reactions were run on an RT-PCR system (ABI7500; Applied Biosystems or Mx3005P; Agilent Technologies). For murine mRNA quantifications, the following primers were used: *Gapdh* primers 5′-CCCACTTGAAGGGTGGAGCCAA-3′ and 5′-TGGCATGGACTGTGGTCATGA-3′; *Il23r* primers 5′-GCTCGGATTTGGTATAAAGG-3′ and 5′-ACTTGGTATCTATGTAGGTAGG-3′; *Il12rb1* primers 5′-ACTGGAATGTGTCTGAAG-3′ and 5′-CGTATCTGGATCTCTTGG-3′; *Il12rb2* primers 5′-CCTCAATGGTATAGCAGAAC-3′ and 5′-TAGCCTTGGAATCCTTGG-3′; *Rorc* primers 5,-AGTCGTCCTAGTCAGAATG-3′ and 5′-ATGTTCCACTCTCCTCTTC-3′; *Il17a* primers 5′-AGGCAGCAGCGATCATCC-3′ and 5′-GTGGAACGGTTGAGGTAGTC-3′; *Il22* primers 5′-CAACTTCCAGCAGCCATAC-3′ and 5′-ATCCTTAGCACTGACTCCTC-3′; *Tbx21* primers 5′-GTTCAACCAGCACCAGAC-3′ and 5′-TCCACCAAGACCACATCC-3′; *Ifng* primers 5′-CTGAGACAATGAACGCTACAC-3′ and 5′-TCCACATCTATGCCACTTGAG-3′; *Il6* primers 5′-CAAGAGACTTCCATCCAG-3′ and 5′-GCATCATCGTTGTTCATAC-3′; *Ccl2* primers 5′-AATGAGATCAGAACCTACAAC-3′ and 5′-TCCTACAGAAGTGCTTGAG-3′; *Tnf* primers 5′-TTCTCATTCCTGTTGTGG-3′ and 5′-ACTTGGTGGTTTGCTACG-3′; *Il1b* primers 5′-GAATCTATACCTGTCCTGTG-3′ and 5′-GTGAAGTCAATTATGTCCTG-3′; *Il10* primers 5′-CTAACCGACTCCTTAATGC-3′ and 5′-AATCACTCTTCACCTGCTC-3′; *Il12a* primers 5′-CCAGGTGTCTTAGCCAGTC-3′ and 5′CTCGTTCTTGTGTAGTTCCAG-3′;, *Il12b* primers 5′-GAATGGCGTCTCTGTCTG-3′ and 5′-GCTGGTGCTGTAGTTCTC-3′;, *Il23* primers 5′-CTGCTTGACTCTGACATC-3′ and 5′-CACTGCTGACTAGAACTC-3′;, *Il18* primers 5′-CAAATGGCCAGTGAACCC-3′ and 5′-AACTCCATCTTGTTGTGTCC-3′. For human mRNA quantification, the following primers were used: *HPRT* primers 5′-TGGCGTCGTGATTAGTGATG-3′ and 5′-CAGAGGGCTACAATGTGATGG-3′; *IL23R* primers 5′- GCCAAGCAGCAATTAAGAAC-3′ and 5′- GACACAGGT TACTTCATCAGG-3′; *IL12RB1* primers 5′- CACAGAGACCCAAGTTAC C-3′ and 5′- GAGGCG AAGAAGATGAGC-3′; *IL12RB2* primers 5′- GTTGGAGTGATTGGAGTG-3′ and 5′- CCTGTGATGTTCTGTGTC-3′. The comparative threshold cycle method and an internal control (*Gapdh* or *HPRT*) were used for normalization of the target genes.

### Induction of Colitis

For colitis experiments, IL-12Rβ2^−/−^.Rag1^−/−^ IL-23R^+/−^.Rag1^−/−^ and IL-23R^−/−^.Rag1^−/−^ mice were injected intraperitoneally with anti-CD40 IgG2a monoclonal antibody, clone FGK45 (BioXCell, West Lebanon, NH) diluted in PBS [17]. 200 µg of anti-CD40 antibody was injected for 20 g of mouse weight. After antibody injection, mice were monitored daily and sacrificed after 7 days or when they were moribund or had lost more than 25% of their initial weight. Serum, liver, lung, heart and intestines were collected at day 7. In some experiments, intestines were collected at day 2. Mice were matched for age, weight and sex in all groups.

### Serum Cytokines

The Cytometric Bead Array Flex Set (BD Biosciences) was used to simultaneously detect IFN-γ, IL-1β, IL-6, IL-12p70, TNF-α, CCL2, and CXCL1 whereas the FlowCytomix (eBioscience) was used to quantify IL-18 and IL-23p19 serum levels according to the respective manufacturers’ instructions and analyzed with a FACS Canto and BD LSRII (BD Bioscience). Standard curves were generated by using reference concentrations supplied by the manufacturers.

### Histopathology Studies

For histopathological examination, samples of the liver, lung, heart and colon were fixed in 10% buffered formalin. After paraffin embedding, 5-µm-wide tissue sections were stained with hematoxylin-eosin according to standard protocol and examined by light microscopy. Criteria for colitis included inflammatory infiltrate of the lamina propria, glandular epithelial hyperplasia and goblet cell depletion. All samples were examined in a blinded fashion.

### Statistics

The data below the detection limit of the assays were given the arbitrary value of zero to allow for statistical analyses. Data were tested for significance using the non-parametric, two-tailed Mann-Whitney *U* test with a minimal threshold of 0.05, in the SPSS 19.0 software.

## Results

### *IL23R* and *IL12RB2* are not Transcribed within the Same Lymphocyte Populations

**Figure 1:**
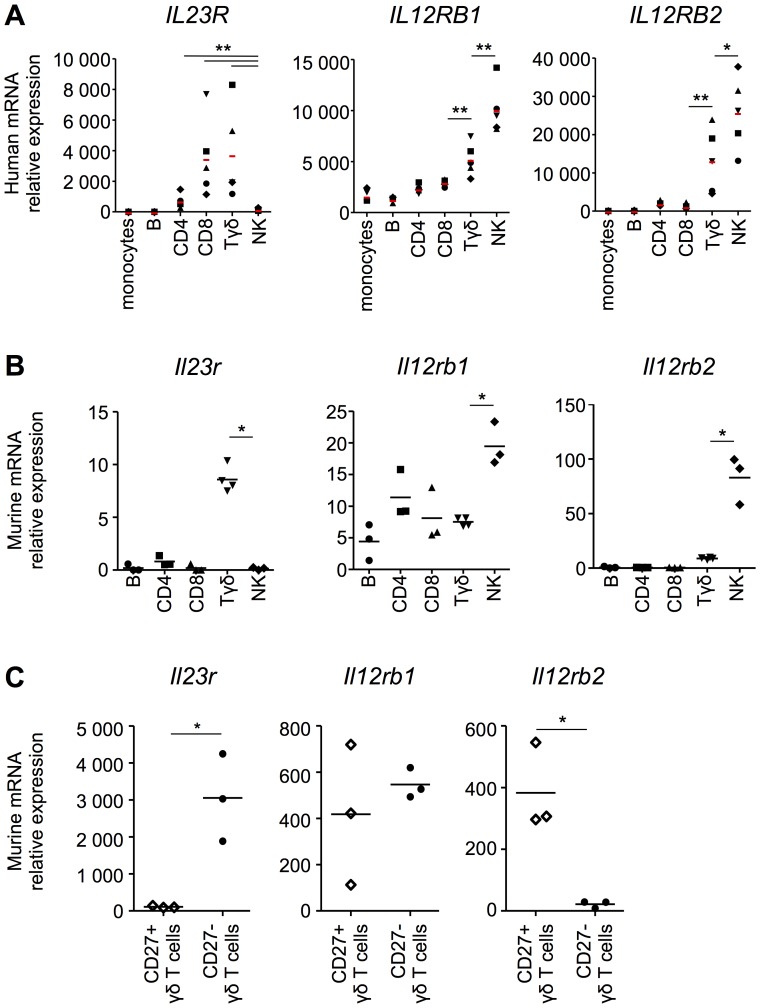
*IL23R* and *IL12RB2* are not transcribed within the same lymphocyte populations. The relative mRNA transcription level of *IL23R*, *IL12RB1* and *IL12RB2* in (A) different sorted PBMC populations of healthy donors and in (B) murine sorted spleen cell populations is shown. (C) The relative mRNA transcription level of *Il23r*, *Il12rb1* and *Il12rb2* in sorted CD27+/− γδ T cell populations from mouse spleens is shown. For all panels, the horizontal bar represents the mean of each group and each symbol represents one sample.*, *p*<0.05, **p<0.01.

As both IL-12 and IL-23 receptors share a common subunit, namely IL-12Rβ1, it has been implied that these receptors are co-expressed. Yet, the pattern of expression of IL-23 and IL-12 receptors is poorly defined. To characterize the expression of these two receptors in different cell populations, we sorted various lymphocytic subsets in PBMC from healthy donors and quantified the level of *IL23R*, *IL12RB1* and *IL12RB2* mRNA. Transcripts for *IL12RB1* were found in all sorted PBMC populations, with lowest levels in B cells and monocytes and highest levels in NK cells (Figure 1A). In contrast, *IL23R* mRNA expression was limited to T cell subsets, with highest expression in both CD8 and γδ T cells, while *IL12RB2* mRNA was expressed in both γδ T cells and NK cells (Figure 1A). The fact that *IL12RB2* mRNA is most abundant in NK cells is consistent with the original name for IL-12, namely natural killer cell stimulatory factor [18], [19]. Most notably, the receptors for IL-12 and IL-23 only appeared to be co-expressed in γδ T cells.

To further explore this unexpected pattern of expression for IL-12 and IL-23 receptors, we opted to examine the mRNA levels of *Il23r*, *Il12rb1* and *Il12rb2* in mouse spleen cells. As for human PBMCs, the mouse spleen presents with a heterogeneous lymphocyte population and is the secondary lymphoid organ best representing cellular populations found in the blood. The mRNA profiles of *Il23r*, *Il12rb1* and *Il12rb2* show a similar transcription pattern to that of human PBMCs in lymphoid cell subsets isolated from mouse spleens. Namely, *Il23r* mRNA is most abundant in γδ T cells and *Il12rb2* is primarily expressed in NK cells, and *Il12rb2* mRNA is also detected at low levels in γδ T cells (Figure 1B). Thus, the patterns of expression of IL-12Rβ1, IL-12Rβ2 and IL-23R are relatively conserved between lymphocytes from human blood and mouse spleen, where only γδ T cells present with detectable levels of all three transcripts coding for the three proteins that constitute the IL-12 and IL-23 receptors.

**Figure 2:**
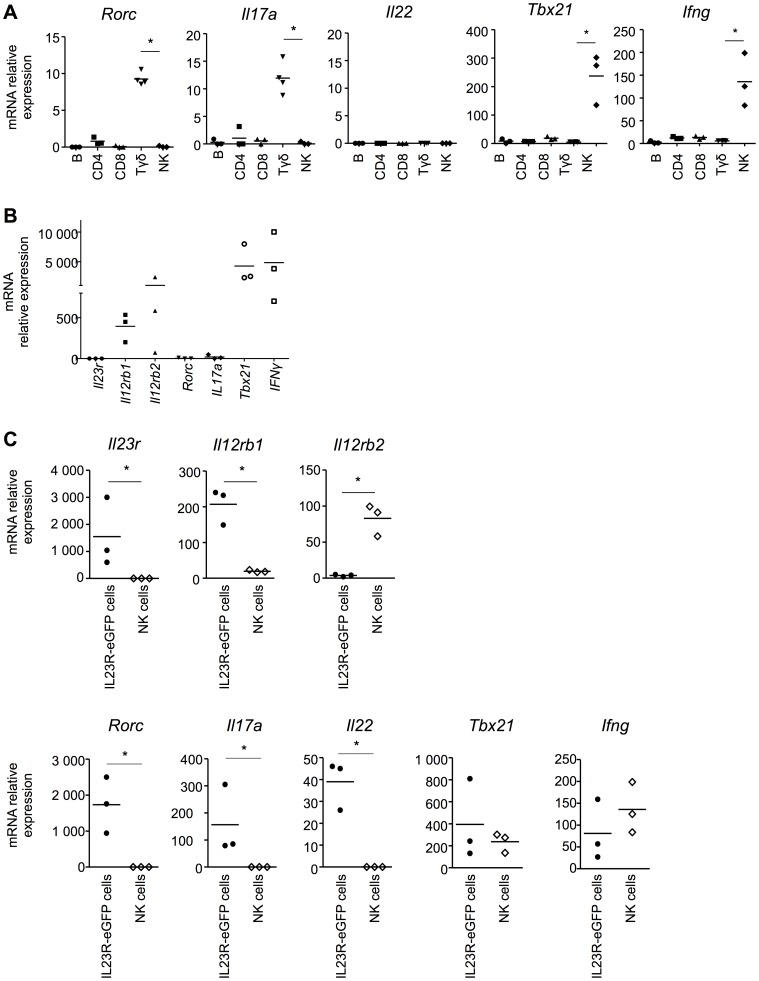
*Il23r* expressing cells exhibit a mixed Th1/Th17 signature profile. (A) The relative mRNA transcription level of *Rorc*, *Il17a*, *Il22*, *Tbx21*, and *Ifng* in sorted murine spleen cells is shown. (B) The relative mRNA transcription level of *Il23r*, *Il12rb1*, *Il12rb2*, *Rorc*, *Il17a*, *Tbx21* and *Ifng* in γδ T cells from RORγt^−/−^ mice is shown. (C) The relative mRNA transcription level of *Il23r*, *Il12rb1*, *Il12rb2*, *Rorc*, *Il17a*, *Il22*, *Tbx21* and *Ifng* in IL23R-eGFP sorted spleen cells and NK cells is shown. For all panels, the horizontal bar represents the mean of each group and each symbol represents one sample.*, *p*<0.05 compared to each group.

Recently, CD27 was shown to efficiently segregate both IL-17- and IFN-γ-producing γδ T cell subsets [20]. As the IL-17 and IFN-γ response are respectively associated with IL-23 and IL-12 pathways, we postulated that the CD27+ and CD27- γδ T cells may exhibit distinct expression patterns of IL-12 and IL-23 receptors, respectively. Indeed, the CD27+ γδ T cells express *Il12rb2* mRNA with no detectable expression of *Il23r* (Figure 1C). Conversely, *Il23r*, but not *Il12rb2*, mRNA was detected in the CD27- γδ T cell subset (Figure 1C). To determine whether this dichotomous expression of *Il12rb2* and *Il23r* reflects Th1 and Th17 signature profiles, we quantified both Th1 and Th17 signature genes in lymphoid subsets. We found that total γδ T cells expressed *Rorc* and *Il17a* mRNA, associated with the Th17 signature and in line with a predominant expression of *Il23r* mRNA in this subset (Figure 2A). In addition, total γδ T cells did not express *Tbx21* nor *Ifng* mRNA, which were instead found to be expressed at high levels in NK cells, in agreement with IL-12Rβ2 expression being associated with a Th1 signature (Figure 2A). Interestingly, *Rorc*, which encodes for the RORγt transcription factor, has been recently suggested to precede *Il23r* mRNA expression, at least in Th17 cells [21]. Hence, we speculated that the IL-17 producing IL23R+ γδ T cell subset would be *Rorc*-dependent. Indeed, γδ T cells from RORγt^−/−^ mice specifically expressed *Il12rb2* mRNA and not *ll23r* (Figure 2B). Moreover, they specifically express the Th1 signature genes, namely *Tbx21* and *Ifng* mRNA (Figure 2B).Taken together, these results favor a model where the receptors for IL-12 and IL-23 are not co-expressed within a given lymphocyte subset and that the expression of these different receptors are driven by different transcription factors.

### IL23R is Not Limited to T Cells

In mice, *Il23r* mRNA expression was only detected in CD27- γδ T cells among the immune cell types tested, whereas in humans both CD8 T cells and γδ T cells carried quantifiable levels of *IL23R* mRNA (Figure 1). To more precisely define the immune cell types expressing the IL-23R, we took advantage of the IL23R-eGFP reporter mouse [16], wherein we sorted all eGFP+ cells from the spleen. Unfortunately, there are currently no reporter mice for *Il12rb2* available. In addition, none of the commercially available antibodies to IL-12Rβ2 provided specific staining. In fact, two distinct antibody clones, namely 305719 from R&D Systems and HAM10B9 from BD Bioscience, non-specifically stained spleen cells from IL12Rβ2^−/−^ mice and did not label NK cells in IL12Rβ2 sufficient mice, precluding a more exhaustive characterization of IL-12Rβ2 expression (Figure S1). We thus opted to compare IL23R-eGFP+ sorted spleen cells with total NK cells, where NK cells serve as a representative cell population bearing high levels of both *Il12rb1* and *Il12rb2* mRNA transcripts and undetectable levels of *Il23r* mRNA transcripts (Figure 1). Expectedly, sorted IL23R-eGFP+ cells express *Il23r* and *Il12rb1*, but not *Il12rb2* mRNA (Figure 2C). As for sorted γδ T cells (Figure 2A), IL23R-eGFP+ cells also express *Rorc* and *Il17a* mRNA (Figure 2C). Surprisingly, however, *Il22*, *Tbx21* and *Ifng* mRNA were detected in IL23R-eGFP+ cells (Figure 2C), but not in γδ T cells (Figure 2A), suggesting that IL23R-eGFP+ cells from the mouse spleen are not exclusively γδ T cells.

**Figure 3:**
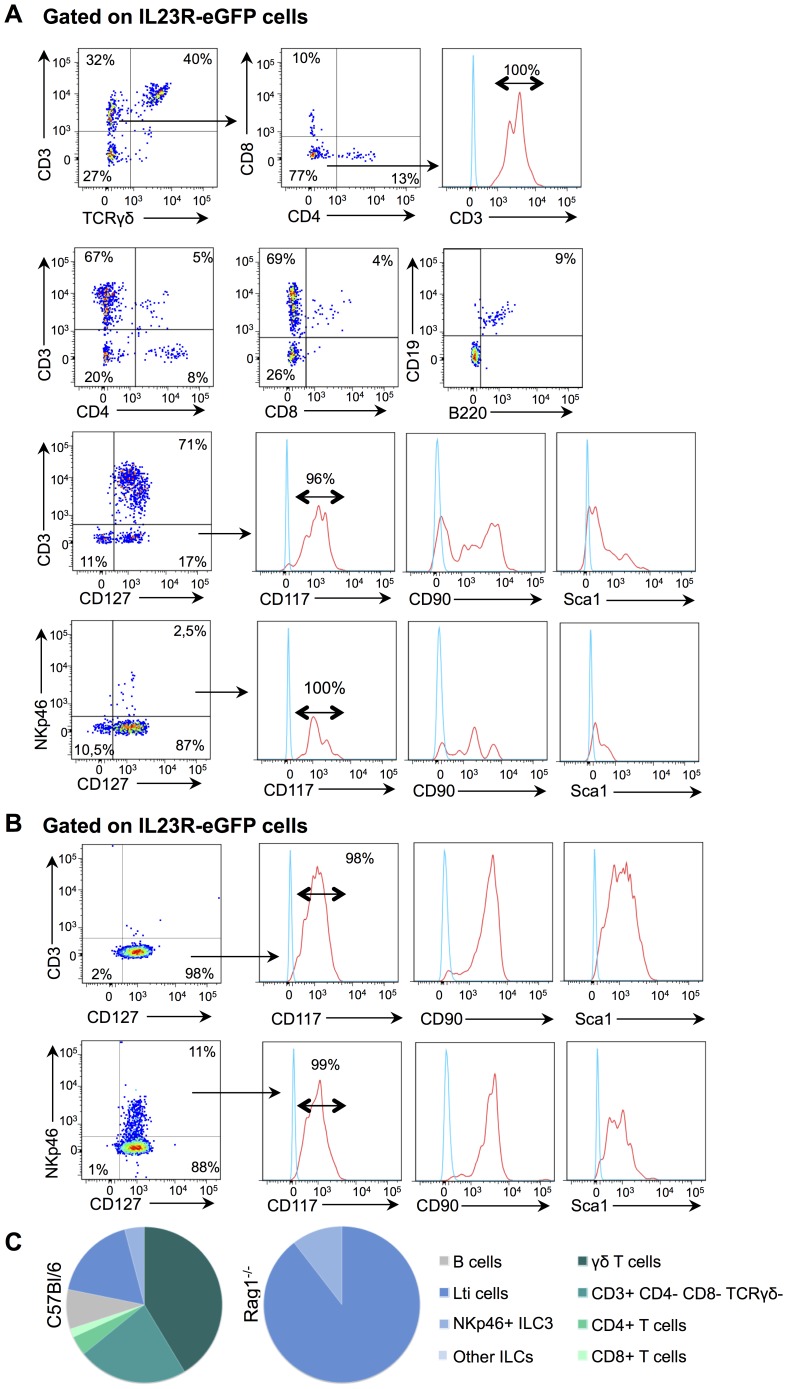
IL-23R is not limited to T cells. (A) Extracellular staining of spleen cells in IL23R-eGFP^+/−^ mice, gated on eGFP positive cells. (B) Extracellular staining of spleen in IL23R^+/−^.Rag1^−/−^ mice, gated on eGFP positive cells. (C) Pie chart representation of subpopulations within the IL23R-eGFP positive cells, in the IL-23R-eGFP^+/−^ mice and IL-23R-eGFP^+/−^.Rag1^−/−^ mice. n = 3.

To characterize these unidentified IL23R+ cells, we performed an exhaustive characterization of IL23R-eGFP+ cells by flow cytometry. In the spleen, IL23R-eGFP+ cells compose less than 0.2% of total cells (Figure S2A). In agreement with the *Il23r* mRNA profiles (Figure 1B), a large proportion of IL23R-eGFP+ cells are γδ T cells, such that they compose approximately 35–40% of all IL23R-eGFP+ cells (Figure 3A). Interestingly, the eGFP signal from the IL23R-eGFP transgene was also detected in a small proportion of both CD4 and CD8 T cells (Figure 3A), though the mRNA level of *Il23r* was undetectable within total CD4 and CD8 T cells (Figure 1B). This is likely due to the fact that fewer than 0,1% of total CD4 or CD8 T cells express IL-23R, while the IL-23R is expressed by more than 7% of total γδ T cells (Figure S2D). This result highlights the enhanced sensitivity of the IL23R-eGFP model for precisely defining the expression pattern of IL-23R. Importantly, regardless of this enhanced sensitivity, NK cells do not express IL-23R, as no IL23R-eGFP+ cells express CD49b, the pan-NK cell marker (Figure S2B).

In addition to γδ T cells as well as CD4 and CD8 αβ T cells, a substantial percentage of IL23R-eGPF+ cells were positive for CD3, although they lacked the expression of CD4, CD8 and γδ TCR (Figure 3A). This phenotype is consistent with the description of both DN T cells and NKT cells, two cell types responding to IL-23 [22], [23]. A small pool of B cells was also found to express IL23R-eGFP (Figure 3A), in agreement with a role for IL-23 in human tonsilar B cell response [24]. The remaining IL-23R expressing cells exhibited the phenotype of innate lymphoid cells, namely CD3- CD127+ CD117+ Lti-like cells and NKp46+ CD127+ CD117+ CD49b- cells (NCR+ ILC3 cells) (Figure 3A) [25]. As innate lymphoid cells compose only a small proportion of total spleen cells, we opted to confirm the expression of IL-23R in these cell subsets by taking advantage of Rag1^−/−^ mice, which lack both B and T cells. In the spleen of Rag1*^−/−^* mice, more than 95% of IL23R-eGFP+ were Lti-like cells and NCR+ ILC3 cells (Figure 3B). NK cells, macrophages, monocytes or granulocytes, did not express IL-23R (Figure S2B, C). However, and unexpectedly, IL-23R was also not expressed on dendritic cells, although IL-23R expression on dendritic cells had been previously reported using an IL-23-Fc fusion protein [26] (Figure S2B, C). Together, these results define IL-23R expressing cells as T cells (mainly γδ T cells), innate lymphoid cells as well as some B cells (Figure 3C). Notably, although Lti-like and ILC3 have mostly been reported in the lymph nodes and mucosal tissues respectively, we find a discrete population of IL-23R+ innate lymphoid cells in the spleen, suggesting that these cells may also contribute to systemic immune responses.

**Figure 4:**
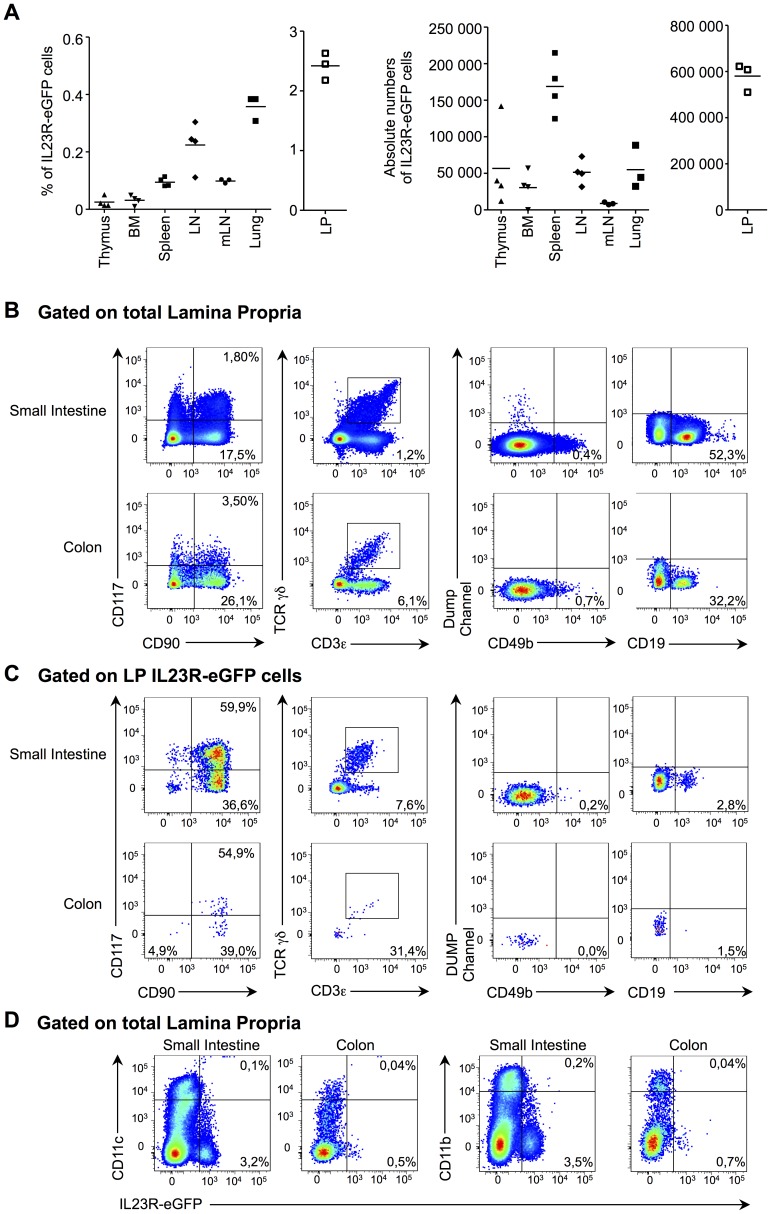
IL-23R+ cells in the intestine are mostly innate lymphoid cells. (A) The percentage and the absolute number of IL-23R-eGFP+ cells in lymphoid organs is depicted. The horizontal bar represents the mean of each group. Each symbol represents data from one mouse. (B) The extracellular staining strategy for the identification of specific immune cellular subsets from total cells in the lamina propria of the small intestine and colon is shown. Electronic gating of viable hematopoietic cells was achieved by using the viable exclusion dye and CD45 expression. γδ T cells (CD3ε+ TCR γδ+), NK cells (CD49b+), B cells (CD19+) and innate lymphoid cells (CD117+CD90+). (C) Identification of IL-23R-eGFP+ immune cell subsets from IL-23R-eGFP^+/−^ mice based on the strategy defined in B. (D) Flow cytometry profiles of IL-23R-eGFP expression in CD11c and CD11b expressing cells. n = 2.

This observation prompted us to examine the distribution of IL-23R+ cells in other lymphoid organs. We found a sizeable proportion and absolute number of IL-23R+ cells in all lymphoid organs examined, where the gut-associated lymphoid tissue exhibited the highest number of IL-23R+ cells (Figure 4A). As the cellular distribution of immune cell types differs considerably between the spleen and the lamina propria of the intestine, we determined the composition of the IL-23R+ cells within this latter tissue. As for the spleen, we found that the IL-23R-eGFP+ cells in the lamina propria of both the small intestine and the colon are comprised of T cells, innate lymphoid cells and B cells (Figure 4B–C). The T cells were mostly γδ T cells and these IL-23R-eGFP+ γδ T cells lacked CD27 expression (Figure S3). However, in contrast with the spleen, the predominant IL-23R+ cell subset in the lamina propria of the gut are innate lymphoid cells (Figure 4B–C). As for the spleen, IL-23R expression was not detected on CD49b+ NK cells. Moreover, neither CD11c+ dendritic cells nor CD11b+ macrophages expressed IL-23R in the lamina of the small intestine and colon (Figure 4D). Together, these results suggest that the distribution of IL-23R+ cells is similar between the spleen and intestinal lymphoid tissue.

### Integration of IL-12Rβ2 and IL-23R Pathways in Systemic Inflammatory Responses

**Figure 5:**
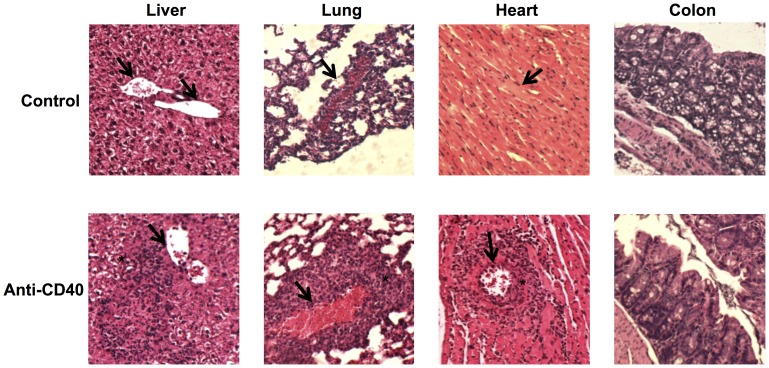
Anti-CD40 injection in Rag-deficient mice leads to multi-organ inflammatory cellular infiltration. Representative H&E microphotographs of liver, lung, heart and colon from C57BL6.Rag1^−/−^ mice are shown. At day 0, mice received 200 µl of saline solution (control, top panels) or 200 µg of anti-CD40 (bottom panels) by intraperitoneal route. Neither inflammatory nor structural changes are observed in the tissues of mice that received the saline solution. The liver, lung and heart of mice treated with anti-CD40 show inflammatory cellular infiltrations (asterisk) near blood vessels (arrows). The colon of anti-CD40 treated mice presents with a mild inflammatory response at the mucosa, a decrease in goblet cells as well as gland arborization.

**Figure 6:**
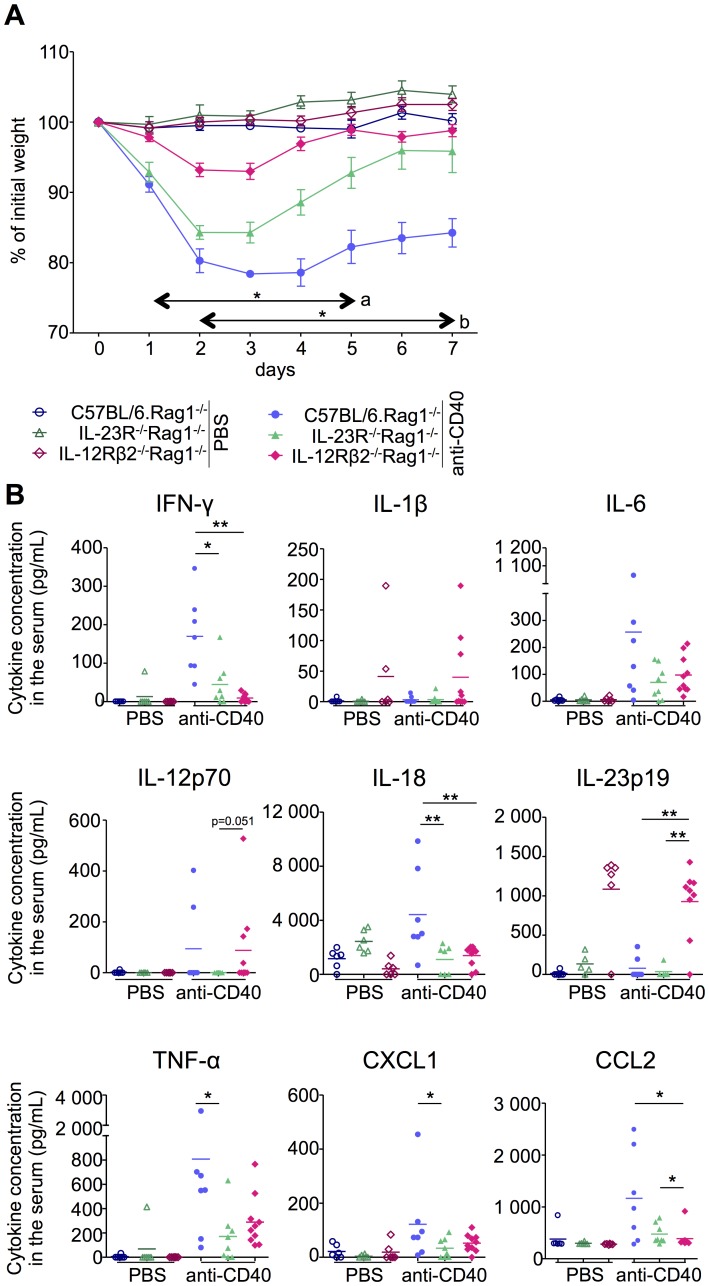
IL-12Rβ2+ and IL-23R+ innate immune cells both contribute to the systemic inflammatory response. C57BL/6.Rag1^−/−^, IL-23R^−/−^.Rag1^−/−^, and IL-12Rβ2^−/−^.Rag1^−/−^ mice were injected with anti-CD40 antibody or PBS (6 to 11 mice per group). (A) Mouse weight relative to the initial weight is shown. a, b p<0.05. (B) Day 7 serum concentrations of IFN-γ, IL-1β, IL-6, IL-12p70, IL-18, IL-23p19, TNF-α, CCL-2 and CXCL1 were quantified. Each symbol represents data for one mouse. *p<0.05, **p<0.01.

The role of the IL-12 and IL-23 pathways has been highlighted in various inflammatory settings, including chronic inflammatory diseases and cancers [27]. As we have defined that IL12Rβ2 and IL23R are expressed by distinct immune cell types (Figure 1–4), we aimed to revisit the unique role of these receptors in an IL-12 and IL-23-dependent inflammatory response. Administration of anti-CD40 antibody in Rag^−/−^ mice provides a model of inflammation dependent on both IL-12 and IL-23 cytokines, and leads to a systemic inflammatory response characterized by the increased levels of various cytokines in the serum [17]. In addition, this anti-CD40-induced systemic inflammatory response leads to cellular infiltration in various tissues, namely the liver, lung, heart and colon (Figure 5). In the Rag^−/−^ setting, only innate lymphoid cells express the IL-23 receptor (Figure 3B), whereas the IL-12 receptor is expressed by NK cells (Figure 1B) reflecting the dichotomous expression profile of these receptors in distinct cell types. We thus generated IL-23R^−/−^.Rag1^−/−^ and IL-12Rβ2^−/−^.Rag1^−/−^ mice, to specifically investigate the contribution of each receptor and their corresponding cell type in the inflammatory response. Intraperitoneal injection of anti-CD40 induces a rapid and significant weight loss in all strains of mice (Figure 6A). This rapid weight loss is indicative of the severity of the systemic inflammatory response, where mice expressing both IL-23 and IL-12 receptors present with the most severe weight loss, while those lacking either the *Il23r* or the *Il12rb2* subunits show only a transient weight loss. Still, IL-23R^−/−^.Rag1^−/−^ mice exhibit a more severe weight loss than IL-12Rβ2^−/−^.Rag1^−/−^ mice. These results thus corroborate the findings that the inflammatory response induced by anti-CD40-treatment is dependent on both IL-12 and IL-23 [17], where the response through the IL-12 receptor largely contributes to the phenotype (Figure 6A). The severity of the weight loss correlates with the serum cytokine levels of IFN-γ and not of other cytokines tested (Figure 6B). Together with the observation that NK cells express the IL-12Rβ2, these results suggest that, upon anti-CD40 treatment, NK cells contribute to the inflammatory response by producing high levels of IFN-γ in response to IL-12. In contrast, although systemic inflammation was apparent in anti-CD40 antibody treated IL-23R^−/−^.Rag1^−/−^ mice, we could not detect IL-12 in the serum. This result suggests that signalling through IL-23R somehow promotes IL-12 production. In mice expressing both IL-12R and IL-23R, IL-12 production in response to IL-23 likely drives an amplification loop, enhancing the severity of the systemic inflammatory response. As neither Lti-like nor NCR+ ILC3 cells have been reported to produce IL-12, additional experiments are required to determine the source of IL-12 production in response to IL-23. Of interest, we also detected serum levels of both IL-1β and IL-23 in IL-12Rβ2^−/−^.Rag1^−/−^ mice, even in the absence of anti-CD40 treatment (Figure 6B). Thus, in non-inflammatory conditions, signalling through IL-12Rβ2 negatively regulates systemic IL-1β production, one of the mediators facilitating the differentiation of IL23R-expressing Th17 cells [28]. Moreover, IL-12 also negatively regulates the basal levels of IL-23. Taken together, we demonstrate an intricate interplay of both IL-12Rβ2 expressing NK cells and IL-23R expressing innate-lymphoid cells in a systemic inflammatory setting.

### Integration of IL-12Rβ2 and IL-23R Pathways in Intestinal Inflammatory Responses

In addition to the IL-12 and IL-23-dependent systemic inflammatory response, anti-CD40 treatment in the Rag^−/−^ setting drives an IL-23-dependent inflammatory response in the intestine [17]. As mentioned above, of all lymphoid tissues tested, the proportion of IL23R+ lymphoid cells is highest in the gut-associated lymphoid tissue (Figure 4A). In the Rag^−/−^ setting, IL-23R expression is limited to innate lymphoid cells (Figure 3), which have been mostly investigated in the context of intestinal inflammatory responses. In this model, we can thus define the role of IL-23R-expressing innate lymphoid cells in the context of intestinal inflammation.

**Figure 7:**
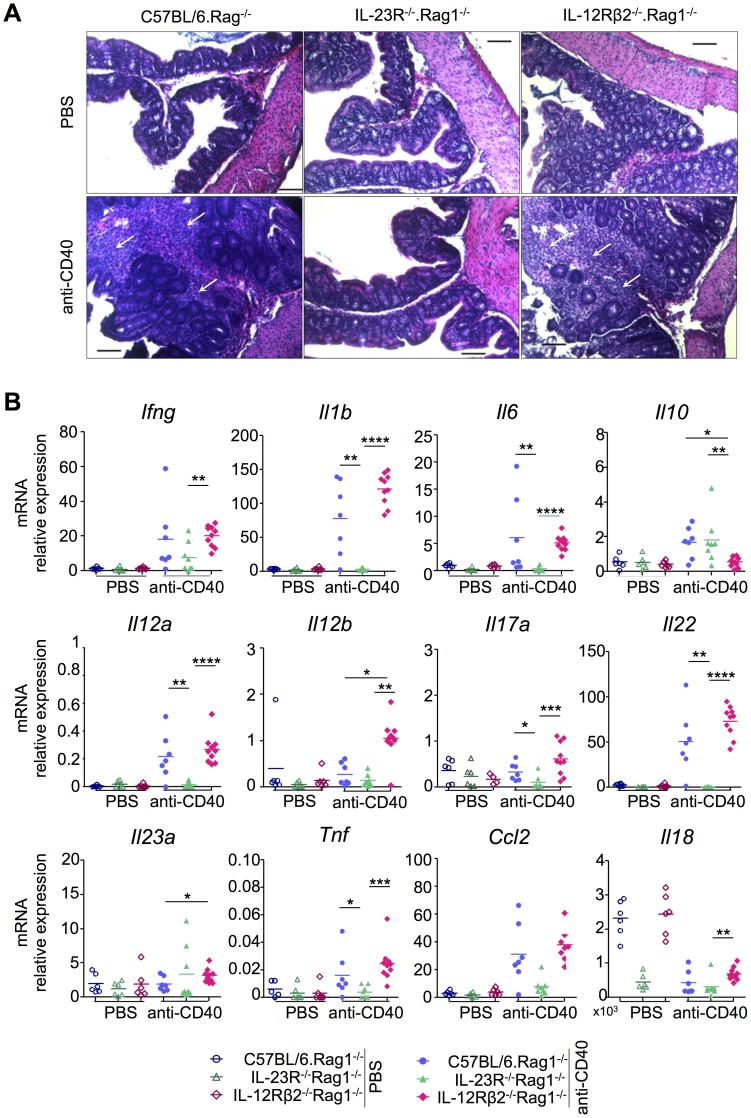
IL-23R+ innate lymphocytes play a dominant role in the intestinal inflammatory response. (A) Histological examination of the colon. Increase in the thickness of the mucosa, inflammatory infiltration (indicated by arrows), goblet cell depletion, discrete glandular hyperplasia and edema of the submucosa were found in C57BL/6.Rag1^−/−^ and IL-12Rβ2^−/−^.Rag1^−/−^ mice which received anti-CD40 antibody. Original magnification 100X. Scale = 100 µm. (B) The relative mRNA transcription levels of cytokines and chemokines in the proximal colon are shown. Each symbol represents data for one mouse. *p<0.05. **p<0.01. ***p<0.001.

**Figure 8:**
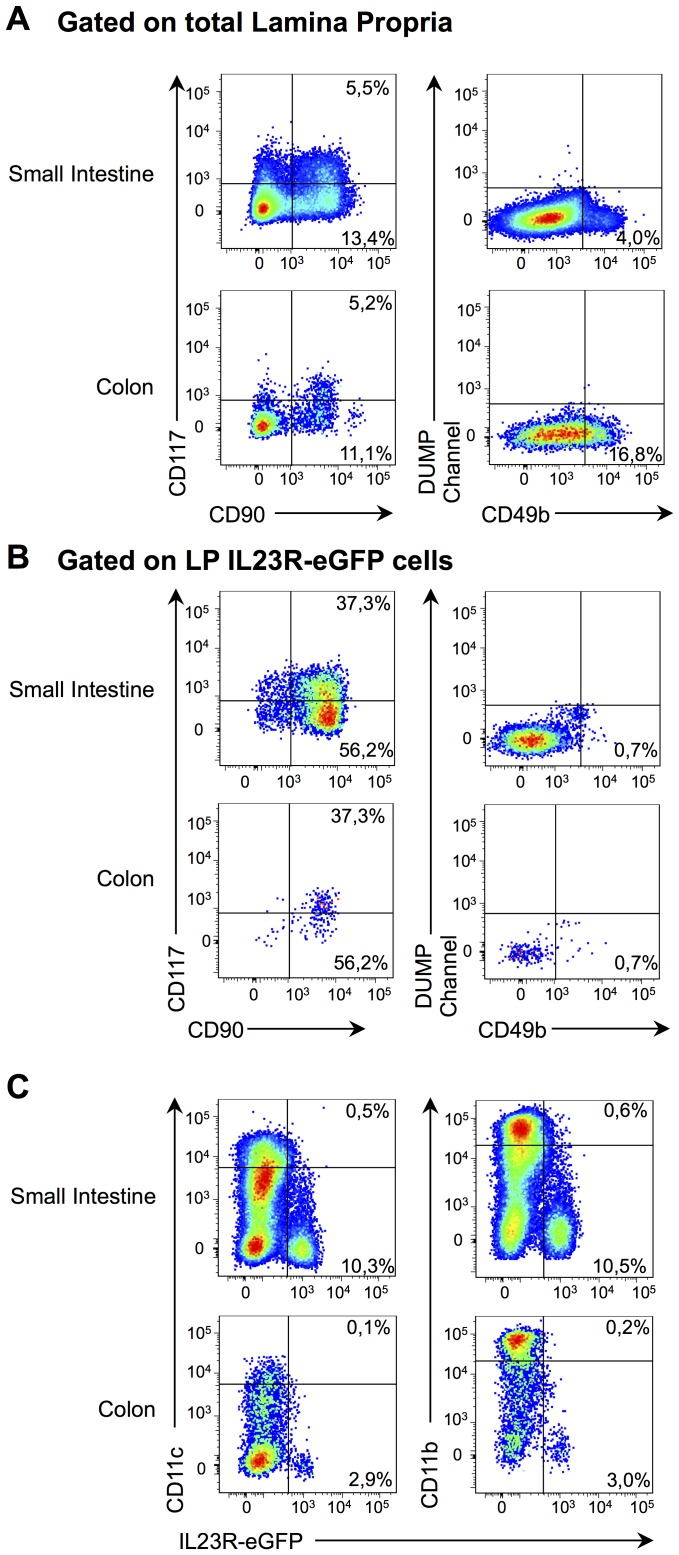
The anti-CD40-induced inflammatory response does not alter the distribution of immune cells in the gut-associated lymphoid tissue. IL-23R-eGFP^−/−^.Rag1^−/−^ mice were treated with anti-CD40 and the intestines were processed at day 2. (A) The extracellular staining strategy for the identification of innate lymphoid cells (CD117+ CD90+) and NK cells (CD49b) within total cells in the lamina propria of the small intestine and colon. Electronic gating of viable hematopoietic cells was achieved by using the viable exclusion dye and CD45 expression. (B) Identification of IL-23R-eGFP+ immune cell subsets from IL-23R-eGFP^−/−^.Rag1^−/−^ anti-CD40-treated mice based on the strategy defined in A. (C) Flow cytometry profiles of IL-23R-eGFP expression in CD11c and CD11b expressing cells. n = 2.

We performed histopathological examinations of intestinal tissues of anti-CD40 treated Rag1^−/−^ mice lacking either IL-12Rβ2 or IL-23R. Mice expressing IL-23R exhibit prominent inflammation in the colon, characterized by a mixed infiltration in the lamina propria, while no cellular infiltration is apparent in IL-23R^−/−^.Rag1^−/−^ mice (Figure 7A). Moreover, following treatment with anti-CD40 antibody, there is a complete absence of induction of mRNA for pro-inflammatory cytokines, namely *Il1b*, *Il6*, *Il12a*, *Il12b*, *Il17a*, *Il18*, *Il22* and *Tnf* in the proximal colon of mice lacking IL-23R. Notably, there was not a complete lack of response in IL-23R^−/−^.Rag1^−/−^ mice, as both *Ifng* and *Il10* mRNA were efficiently induced in the colon (Figure 7B). However, this was insufficient to drive detectable histopathological lesions (Figure 7A). Still, upon anti-CD40 treatment, IL-23R^−/−^.Rag1^−/−^ mice presented with a sizeable proportion of IL-23R+ cells in the lamina propria of the small intestine and the colon, which was mostly comprised on innate lymphoid cells (Figure 8A, B). Remarkably, IL-23R expression was still not observed on dendritic cells and macrophages in this experimental setting (Figure 8C).

In contrast to IL-23R^−/−^.Rag1^−/−^ mice, IL-12Rβ2^−/−^.Rag1^−/−^ mice exhibited elevated mRNA levels of most pro-inflammatory cytokines. Indeed, we surprisingly observed elevated *Ifng* mRNA levels in the colon (Figure 7B), although we had not detected IFN-γ in the serum of these mice upon anti-CD40 treatment (Figure 6B). This result clearly indicates an IL-12-independent pathway for local IFN-γ production in intestinal inflammation. As IL-18 has been shown to promote IFN-γ production, we investigated the regulation of *Il18* mRNA expression [29]. Upon anti-CD40 treatment, *Il18* mRNA was highest in IL-12Rβ2^−/−^.Rag1^−/−^ mice relative to the other strains, suggesting that IL-18 may promote IFN-γ production in this setting (Figure 7B). However, *Il18* mRNA expression in anti-CD40-treated IL-12Rβ2^−/−^.Rag1^−/−^ mice was much lower than at steady state levels, likely due to the destruction of epithelial cells, which constitutively express *Il18* mRNA [30] (Figure 7B). In addition, *Il18* mRNA levels were severely compromised in IL-23R^−/−^.Rag1^−/−^ mice, even in the non-inflammatory setting (Figure 7B), suggesting that basal IL-23 response promotes the constitutive expression of *Il18* mRNA, adding to the already complex regulation of *Il18* mRNA levels [31]. Additional studies are required to define whether IL-18 is truly responsible for the enhanced IFN-γ production in the IL-12Rβ2^−/−^.Rag1^−/−^ mice upon anti-CD40 treatment.

Finally, as IL-12Rβ2 and IL-23R are respectively expressed by NK cells and innate lymphoid cells in the Rag-deficient setting, we chose to compare the relative proportion of these cell subsets in the lamina propria of the small intestine of C57BL/6.Rag1^−/−^, IL-23R^−/−^.Rag1^−/−^ and IL-12Rβ2^−/−^.Rag1^−/−^ mice that had received anti-CD40 treatment. We found that the proportion and absolute number of NK cells did not vary between the three strains, further suggesting that NK cells play little role in the local inflammatory response of the gut (Figure S4). In contrast, IL-12Rβ2^−/−^.Rag1^−/−^ mice presented with a significantly larger proportion and number of innate lymphoid cells than both C57BL/6.Rag1^−/−^ and IL-23R^−/−^.Rag1^−/−^ (Figure S4), providing further credence to the view that the IL-12 pathway negatively regulates the IL-23-pathway. Moreover, this result suggests that IL-12 may mediate its effect by modulating the recruitment or accumulation of innate lymphoid cells to the inflammatory site.

Taken together, these findings demonstrate that IL-23R-expressing innate lymphoid cells play a key role in promoting intestinal inflammation through the production of pro-inflammatory cytokines, while IL-12Rβ2-expressing cells negatively regulate this process.

## Discussion

The IL-23 and IL-12 pathways are intimately linked since these cytokines share a protein subunit, IL-12p40, and their respective receptors also share a common receptor chain, IL-12Rβ1 [32]. The specific role of these cytokines has been elucidated, at least in part, using IL-12p35, IL-12p40 and IL-23p19 deficient mice [33]. Yet, the expression profiles of IL-23 and IL-12 receptors have not been extensively studied [34]. In particular, few studies have directly addressed IL-12Rβ2 subunit protein expression [35]–[37]. Recently, an IL-23R reporter mouse has allowed a more accurate depiction of IL-23R expression on various cell subsets, although a thorough characterization of IL-23R expression in different innate immune cell types had not been performed [16]. Moreover, while they share the IL-12Rβ1 subunit for expression and function, IL-12Rβ2 and IL-23R co-expression has not been investigated. We have thus decided to define the coordinated IL-12Rβ2 and IL-23R expression on immune cell subsets.

Using the IL-23ReGFP reporter mice, we have exhaustively characterized the immune cell types expressing IL-23R. Unfortunately, due to lack of a similar genetic tool and to the absence of IL-12Rβ2-specific antibody, the characterization of IL-12Rβ2 expression remains limited to qPCR analysis of sorted cell subset. Regardless, our data strongly support the view that the receptors for IL-12 and IL-23 are not co-expressed; rather they are expressed by distinct cell types in mice. This dichotomous pattern of expression for IL-12 and IL-23 receptors will need to be validated by flow cytometry on human cell subsets as the appropriate antibodies become available. Regardless, the pattern of receptor expression appears mostly conserved between human and mouse. Of interest, human but not mouse CD8 T cells express detectable levels of mRNA, suggesting that a greater proportion of human CD8 T cells express the IL-23R relative to mouse CD8 T cells. The reason for this discrepancy remains unclear but may be of biological relevance when comparing IL-23-dependent inflammatory responses in both mouse and human. Still, the dichotomous expression pattern of the receptors for IL-12 and IL-23, at least in mice, suggests that the expression of both *Il12rb2* and *Il23r* are tightly regulated by distinct (and maybe opposing) transcription factors. To that effect, we find that IL-12Rβ2 expressing NK cells exhibit a Th1-like signature, while IL-23R expressing cells (which include Lti-like cells, T cell subset as well as a low number of B cells) exhibit a Th17-like profile, suggesting that transcription factors driving IL-12Rβ2 and IL-23R maybe key regulators of Th1 and Th17 signatures, respectively. In addition, RORγt^−/−^ mice lack IL-23R expressing γδ T CD27- cells. Interestingly, *Rorc* appears to be essential in driving the differentiation of Lti cells [38]. Still, it remains to be seen whether *Rorc* is required for the expression of IL23R or whether it acts upstream in the differentiation of IL-23R^+^ cells. Similarly, the specific transcription factor(s) driving IL-12Rβ2 expression has yet to be identified.

Notably, we could not detect IL-23ReGFP expression in NK cells. Yet, in contrast with our findings, NK cells, characterized as NK1.1^+^CD3^−^ cells, were shown to modestly produce IL-22, but not IL-17, when cultured in the presence of IL-23 [39]. One possible explanation that may help reconcile these two findings, is that the NK1.1^+^CD3^−^ cells may include a subset of ILC3 cells that are responsive to IL-23 [40], [41]. This issue will likely be resolved as more tools become available to study IL-12Rβ2 expression. To that effect, the commercially available antibodies apparently directed to IL-12Rβ2 exhibit non-specific staining in IL-12Rβ2-deficient mice. However, they have been used even in recent publications to seemingly define IL-12Rβ2 expression on various cellular subsets [42]–[46]. Similarly, we demonstrate that IL-23R is not expressed on dendritic cells, although a previous report using an IL-23-Fc fusion protein suggested otherwise [26]. We would like to caution investigators using these and other reagents, for which a thorough validation has not been performed.

The contribution of IL-23R to inflammatory bowel diseases has been explored in various mouse models of colitis, sometimes demonstrating a protective role [47] and other times exacerbating disease [48], [49]. Our data strongly supports a pathogenic role for IL-23R+ innate lymphoid cells specifically in the local innate immune response [39], [48], [50]. This is in line with previous work showing that both anti-CD90 (Thy-1) depletion in Rag1-deficient mice or use of Rag1.Rorc-double deficient mice, which principally lack IL-23R+ innate lymphoid cells, are similarly resistant to anti-CD40-induced colitis [48]. Of note, although anti-CD90 antibodies have now been used in various experimental settings to deplete innate lymphoid cells [47], [48], [51], CD90 expression is also found on a sizeable proportion of NK cells (Figure S5). Interestingly, a protective role for innate lymphoid cells has mostly been observed in the context of anti-CD90 depletion, suggesting that the CD90-expressing NK cells may confer at least part of this protective role, whereas IL-23R+ innate lymphoid cells participate more actively in the local inflammatory responses.

Importantly, genome wide association studies in human inflammatory bowel disease also suggest a prominent role of the innate immune system and of IL-23R variants in defining susceptibility to disease [6], [52]–[56]. Our results are in agreement with the view that innate immune cells expressing IL-23R contribute to the pathology by producing pro-inflammatory cytokines [50], [57]. Specific targeting of IL-23 response in innate immune cells in the gut may thus provide a therapeutic benefit to patients suffering from inflammatory bowel disease.

In contrast, targeting IL-12R may be most relevant for the control of systemic inflammatory responses and, by its negative regulatory role on IL-23 responses, may actually exacerbate the local inflammatory responses. In fact, IL-12Rβ2^−/−^ mice eventually develop systemic autoimmune responses, characterized by the presence of anti-nuclear autoantibodies [58]. Moreover, these mice exhibit an increased propensity to develop cancers [58]. As *Il12rb2* mRNA is abundantly expressed in NK cells, it is tempting to suggest that lack of IL-12Rβ2 expression is sufficient to deregulate NK cell immune surveillance, thereby increasing cancer prevalence.

The complexity of these two pathways, namely IL-12 and IL-23, is further highlighted in the anti-CD40 inflammatory model. Indeed, we intriguingly find detectable IL-12 serum levels only when IL-23R is expressed. This suggests that the response to IL-23 promotes IL-12 production, highlighting an intricate interplay between the two cytokines in promoting an inflammatory response. In contrast, we detected basal serum levels of IL-1 and IL-23 in IL-12Rβ2-deficient mice, even in steady state conditions. This result suggests that the continuous response to IL-12 in non-inflammatory conditions inhibits IL-1 production, known to drive IL-23 responses. As such, at steady state, IL-12 may serve to control inflammation.

In conclusion, our data reveal a dichotomous expression of the IL-12Rβ2 and IL-23R which help to explain the contribution of the IL-12 and IL-23 pathways to distinct immune pathologies. In addition, the lack of IL-12 and IL-23 receptor co-expression on a given cell strongly suggests that IL-12 and IL-23 cytokines are not involved in modulating the nature of the immune response by changing the transcription profile of the responding cell. Instead, and similar to what has been described in the context of Th1 and Th17 [59], IL-12 and IL-23 cytokines may specifically maintain the survival or enhance cytokine production of cells already expressing their respective receptor and the corresponding transcription profile. Our work also highlights an intricate interplay of these two pathways in the cellular immune response in the context of both a systemic and local inflammatory setting. A better understanding of the dynamic regulation of IL-12Rβ2 and IL-23R in various inflammatory contexts is warranted to guide the design of novel and specific therapeutic approaches in inflammatory diseases.

## Supporting Information

Figure S1 **Commercially available antibodies to IL-12Rβ2 provide non-specific staining.** (A) Representative profile of APC-conjugated anti-IL-12Rβ2 antibody (clone 305719 from R&D Systems) staining in total spleen cells from IL23R-eGFP+/− mice (control, left panel) and IL-12Rβ2−/− mice (right panel). Similar results were obtained with both the PE and APC conjugated antibodies. (B) Representative profile of Pacific Blue conjugated anti-CD49b antibody (clone DX5 from Biolegend), unconjugated anti-IL12Rβ2 antibody (clone HAM10B9 from BD Biosciences) followed by PE-conjugated anti-hamster IgG (clones G70-204, G94-90.5 from BD Biosciences) in total spleen cells from IL23R-eGFP+/− mice (control, left panel) and IL-12Rβ2−/− mice (right panel).
(TIF)

Figure S2 **NK cells, dendritic cells, macrophages and granulocytes do not express IL-23R.** (A) A representative plot presenting the percentage of IL23R-eGFP+ cells in the spleen. (B) The expression of NK1.1, NKp46, CD49b, CD11c, CD11b, mouse pDC antigen-1 (mPDCA-1), CD8α, and Gr1 on IL23R-eGFP positive cells from the spleen of IL23R-eGFP+/− mice is shown. (C) Gating strategy for the extracellular staining of total spleen cells which was applied in B. (D) Percentage of IL23R-eGFP positive cells among B cells, CD4+ T cells, CD8+ T cells, CD3+ CD4- CD8- TCR γδ- T cells, γδ T cells, Lti-like cells, and NCR+ ILC3 cells in the spleen of IL23R-eGFP+/− mice. Each symbol represents data from one mouse. The horizontal bar represents the mean of each group.
(TIF)

Figure S3 **IL-23R-eGFP+ γδ T cells are CD27-.** Flow cytometry profiles of γδ T cell subsets based on CD27 and IL-23R-eGFP expression is shown for the spleen, the lamina propria of the small intestine (SI LP) and the lamina propria of the colon (Colon LP). n ≥ 2.
(TIF)

Figure S4 **Relative distribution of NK cells and innate immune cells.** The proportion and absolute number of NK cells and innate immune cells from the lamina propria of the small intestine of day 2 anti-CD40-treated C57BL/6.Rag1−/−, IL-23R-eGFP−/−.Rag1−/−, IL-12Rβ2−/−.Rag1−/− mice, is shown. Note the increased number of innate immune cells in IL-12Rβ2−/−.Rag1−/− mice. The data represents the mean value of two to three mice per group performed in three independent experiments.
(TIF)

Figure S5 **A subset of NK cells expresses the CD90 (Thy-1) antigen.** CD90 expression is shown on NK cells (CD49b+) of (A) the lamina propria of the small intestine (SI LP) and the lamina propria of the colon (Colon LP) for IL-23R-eGFP+/− mice and (B) the spleen, the lamina propria of the small intestine (SI LP) and the lamina propria of the colon (Colon LP) for C57BL/6.Rag1−/− mice. The intestines of C57BL/6.Rag1−/− mice treated with anti-CD40 were processed at day 2. n = 2.
(TIF)
