# Supplementary figures and images for: The Dichotomous Pattern of IL-12R and IL-23R Expression Elucidates the Role of IL-12 and IL-23 in Inflammation
Source: PLoS One. 2014 Feb 21;9(2):e89092. doi: 10.1371/journal.pone.0089092 (PMC3931659; doi:10.1371/journal.pone.0089092)

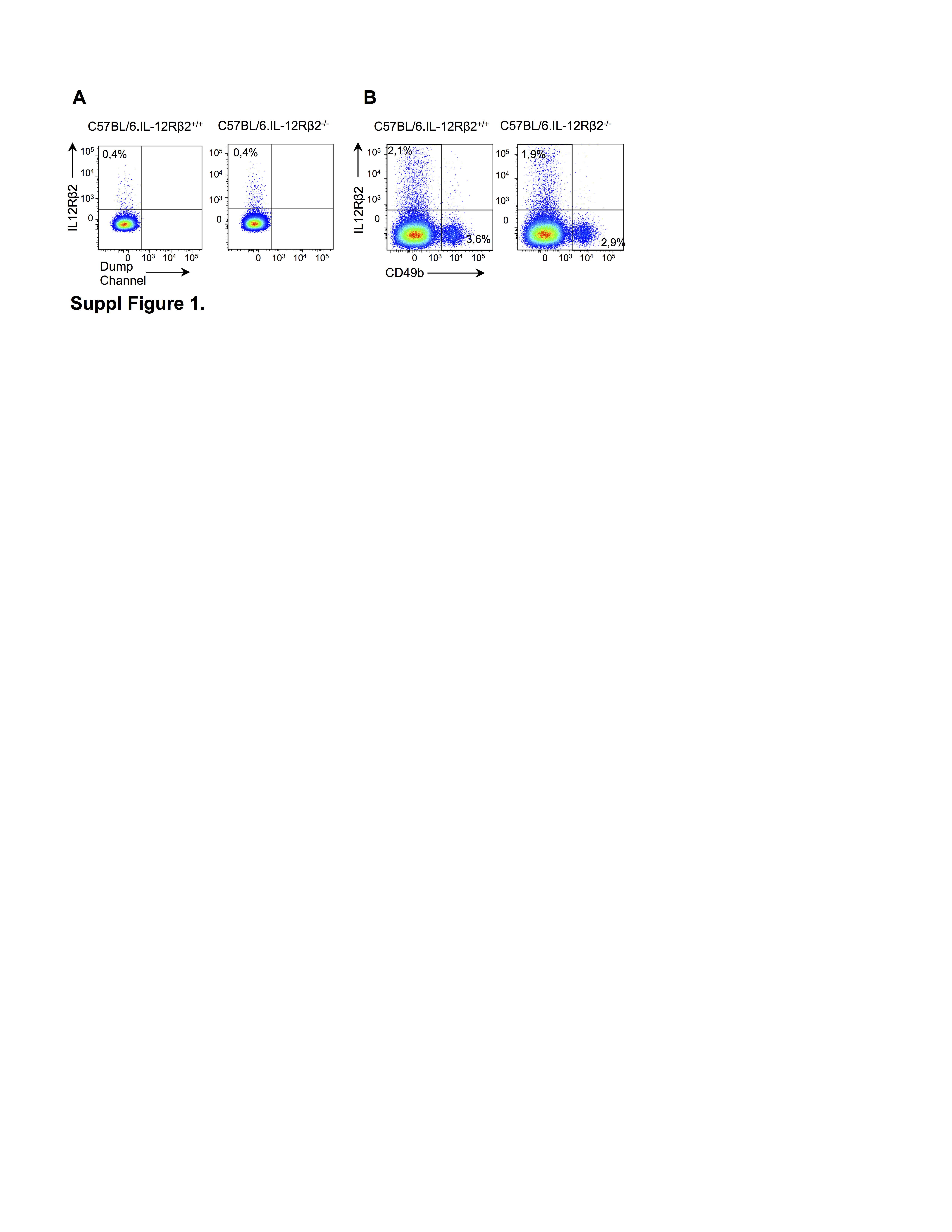

Supplement: Figure S1 — Commercially available antibodies to IL-12Rβ2 provide non-specific staining. (A) Representative profile of APC-conjugated anti-IL-12Rβ2 antibody (clone 305719 from R&D Systems) staining in total spleen cells from IL23R-eGFP+/− mice (control, left panel) and IL-12Rβ2−/− mice (right panel). Similar results were obtained with both the PE and APC conjugated antibodies. (B) Representative profile of Pacific Blue conjugated anti-CD49b antibody (clone DX5 from Biolegend), unconjugated anti-IL12Rβ2 antibody (clone HAM10B9 from BD Biosciences) followed by PE-conjugated anti-hamster IgG (clones G70-204, G94-90.5 from BD Biosciences) in total spleen cells from IL23R-eGFP+/− mice (control, left panel) and IL-12Rβ2−/− mice (right panel). (TIF) [file pone.0089092.s001.tif]

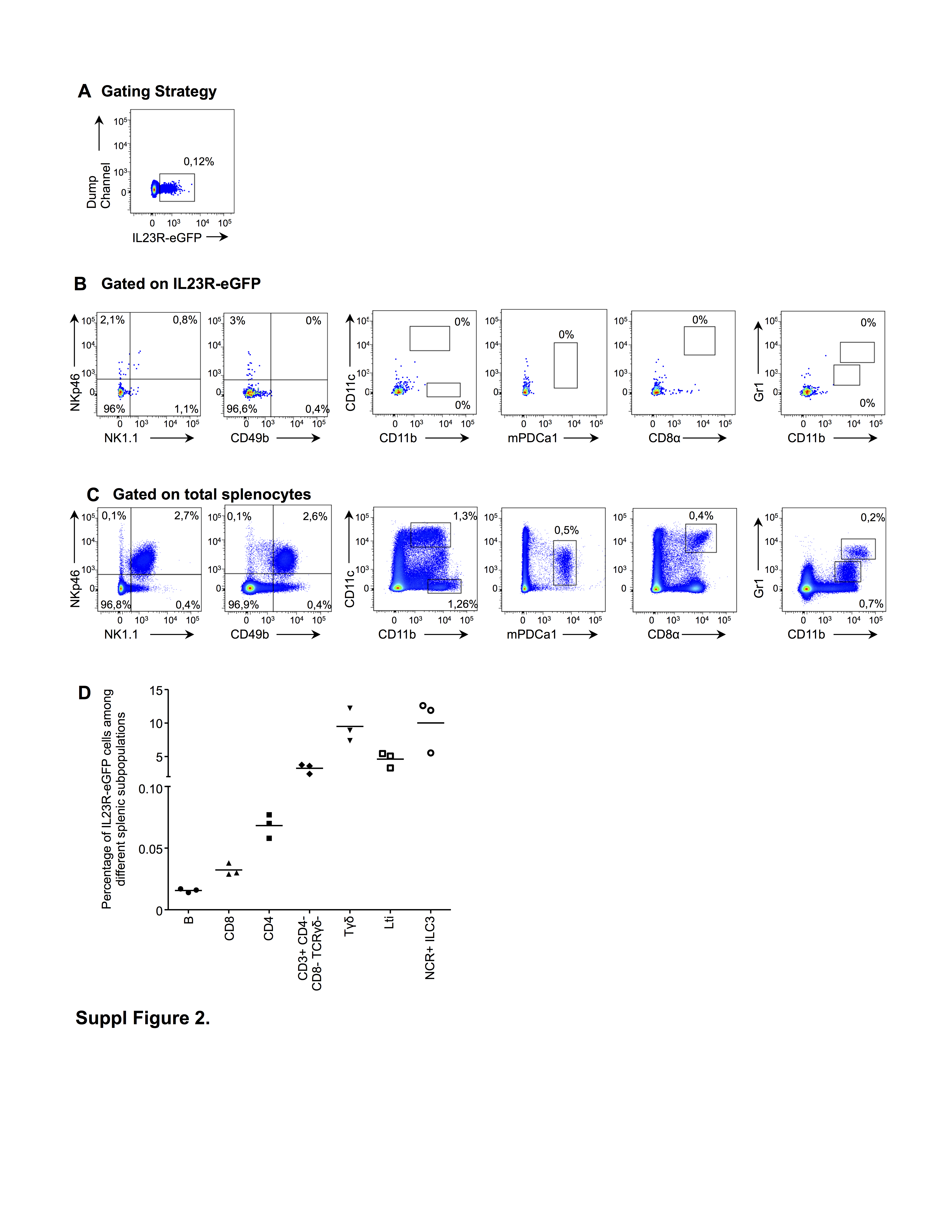

Supplement: Figure S2 — NK cells, dendritic cells, macrophages and granulocytes do not express IL-23R. (A) A representative plot presenting the percentage of IL23R-eGFP+ cells in the spleen. (B) The expression of NK1.1, NKp46, CD49b, CD11c, CD11b, mouse pDC antigen-1 (mPDCA-1), CD8α, and Gr1 on IL23R-eGFP positive cells from the spleen of IL23R-eGFP+/− mice is shown. (C) Gating strategy for the extracellular staining of total spleen cells which was applied in B. (D) Percentage of IL23R-eGFP positive cells among B cells, CD4+ T cells, CD8+ T cells, CD3+ CD4- CD8- TCR γδ- T cells, γδ T cells, Lti-like cells, and NCR+ ILC3 cells in the spleen of IL23R-eGFP+/− mice. Each symbol represents data from one mouse. The horizontal bar represents the mean of each group. (TIF) [file pone.0089092.s002.tif]

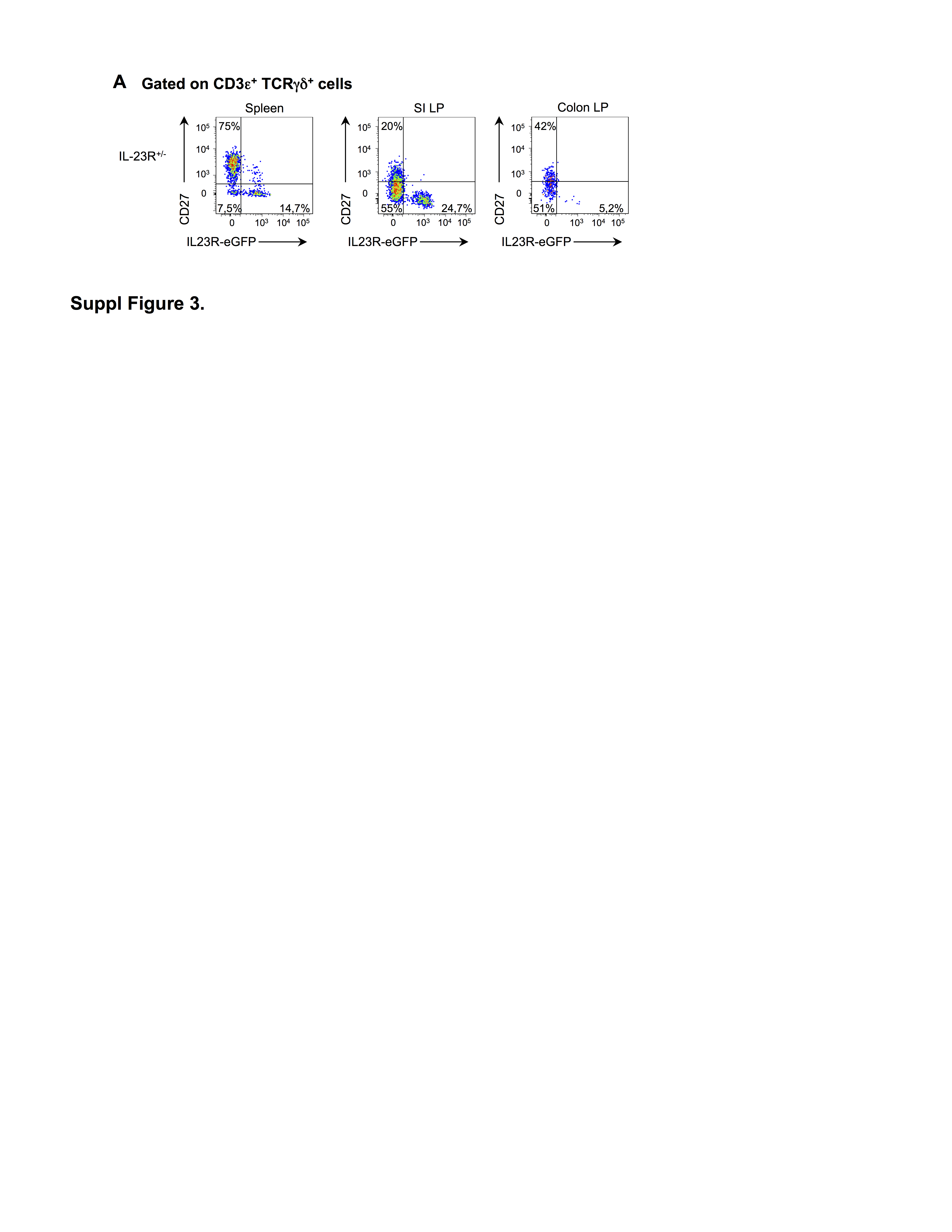

Supplement: Figure S3 — IL-23R-eGFP+ γδ T cells are CD27-. Flow cytometry profiles of γδ T cell subsets based on CD27 and IL-23R-eGFP expression is shown for the spleen, the lamina propria of the small intestine (SI LP) and the lamina propria of the colon (Colon LP). n ≥ 2. (TIF) [file pone.0089092.s003.tif]

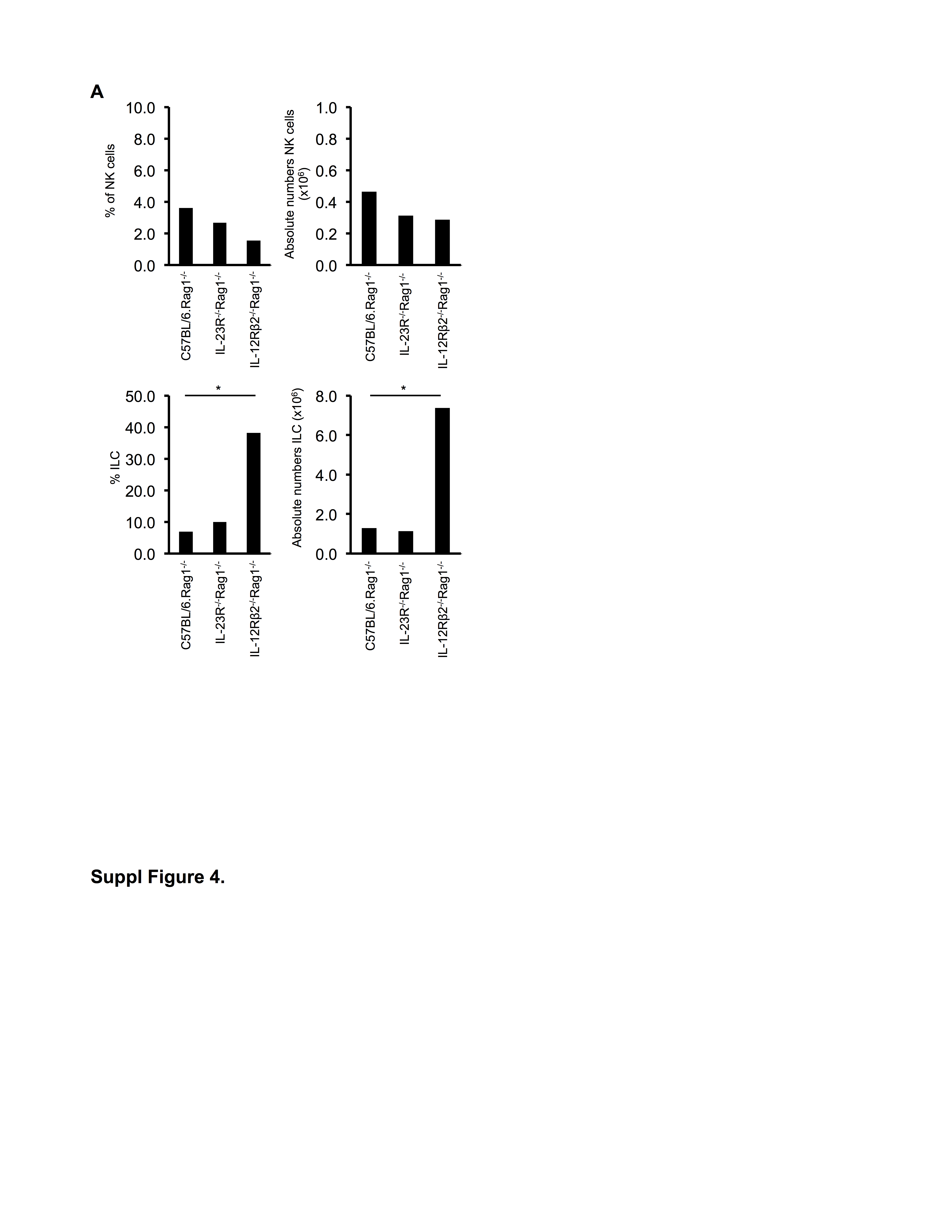

Supplement: Figure S4 — Relative distribution of NK cells and innate immune cells. The proportion and absolute number of NK cells and innate immune cells from the lamina propria of the small intestine of day 2 anti-CD40-treated C57BL/6.Rag1−/−, IL-23R-eGFP−/−.Rag1−/−, IL-12Rβ2−/−.Rag1−/− mice, is shown. Note the increased number of innate immune cells in IL-12Rβ2−/−.Rag1−/− mice. The data represents the mean value of two to three mice per group performed in three independent experiments. (TIF) [file pone.0089092.s004.tif]

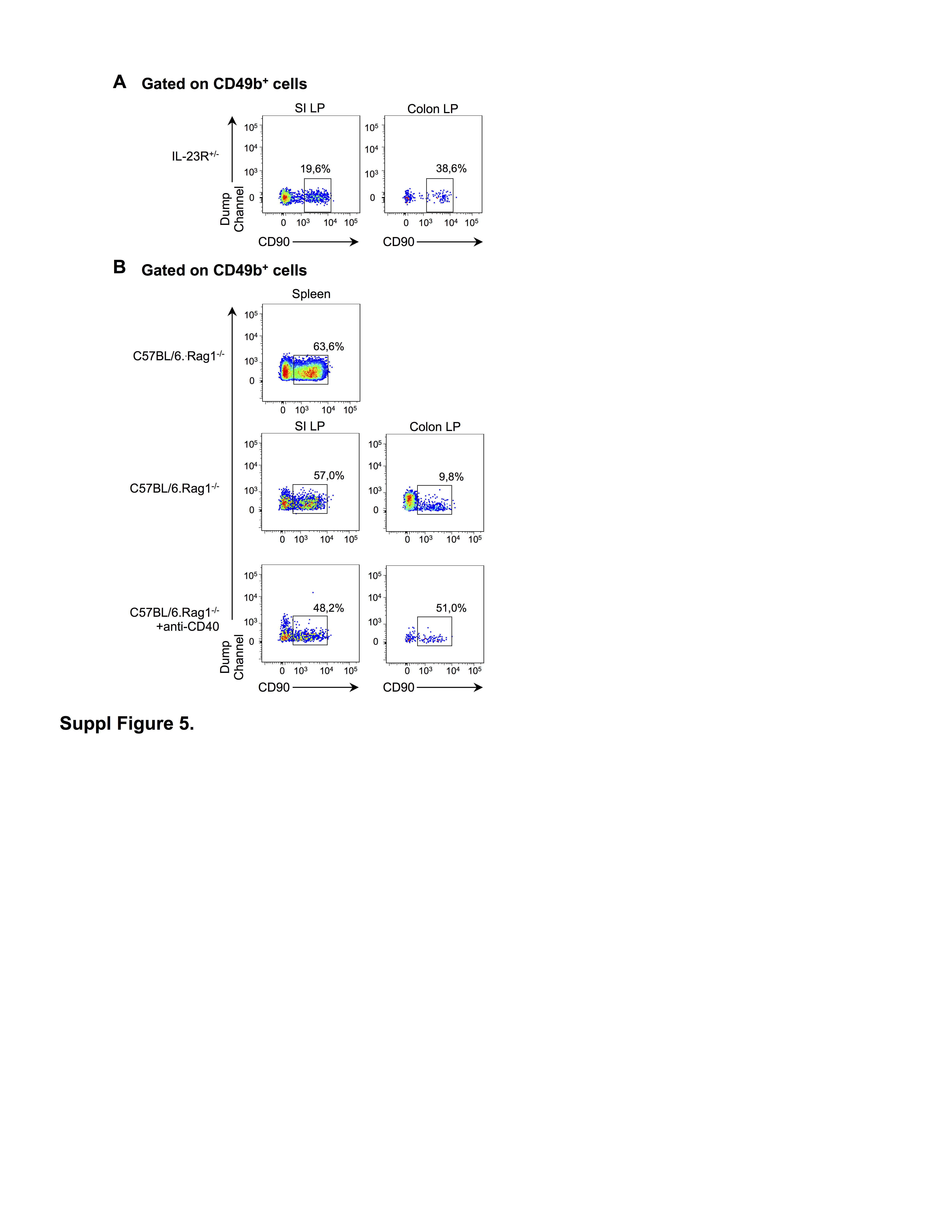

Supplement: Figure S5 — A subset of NK cells expresses the CD90 (Thy-1) antigen. CD90 expression is shown on NK cells (CD49b+) of (A) the lamina propria of the small intestine (SI LP) and the lamina propria of the colon (Colon LP) for IL-23R-eGFP+/− mice and (B) the spleen, the lamina propria of the small intestine (SI LP) and the lamina propria of the colon (Colon LP) for C57BL/6.Rag1−/− mice. The intestines of C57BL/6.Rag1−/− mice treated with anti-CD40 were processed at day 2. n = 2. (TIF) [file pone.0089092.s005.tif]
